# TULP2 deletion mice exhibit abnormal outer dense fiber structure and male infertility

**DOI:** 10.1002/rmb2.12467

**Published:** 2022-05-23

**Authors:** Yuki Oyama, Haruhiko Miyata, Keisuke Shimada, Tamara Larasati, Yoshitaka Fujihara, Masahito Ikawa

**Affiliations:** ^1^ Graduate School of Pharmaceutical Sciences Osaka University Suita Japan; ^2^ Department of Experimental Genome Research Research Institute for Microbial Diseases Osaka University Suita Japan; ^3^ Graduate School of Medicine Osaka University Suita Japan; ^4^ 13875 Department of Bioscience and Genetics National Cerebral and Cardiovascular Center Suita Japan; ^5^ The Institute of Medical Science The University of Tokyo Tokyo Japan; ^6^ Center for Infectious Disease Education and Research Osaka University Osaka Japan

**Keywords:** flagellum, outer dense fiber, sperm motility, spermiogenesis, tubby‐like protein 2

## Abstract

**Purpose:**

*Tulp2* (tubby‐like protein 2) is a member of the tubby protein family and expressed predominantly in mouse testis. Recently, it was reported that *Tulp2* knockout (KO) mice exhibited disrupted sperm tail morphology; however, it remains to be determined how TULP2 deletion causes abnormal tail formation.

**Methods:**

The authors analyzed male fertility, sperm morphology, and motility of two *Tulp2* KO mouse lines that were generated using the conventional method that utilizes homologous recombination in embryonic stem (ES) cells as well as the clustered regularly interspaced short palindromic repeats/CRISPR‐associated protein 9 (CRISPR/Cas9) system. Furthermore, the authors observed the spermatogenesis of *Tulp2* KO mice in more detail using scanning and transmission electron microscopy (SEM and TEM).

**Results:**

Both mouse lines of *Tulp2* KO exhibited male infertility, abnormal tail morphology, and impaired sperm motility. No overt abnormalities were found in the formation of the mitochondrial sheath in *Tulp2* KO mice using the freeze‐fracture method with SEM. In contrast, abnormal outer dense fiber (ODF) structure was observed in *Tulp2* KO testis with TEM.

**Conclusions:**

TULP2 may play roles in the correct formation and/or maintenance of ODF, which may lead to abnormal tail morphology, impaired sperm motility, and male infertility.

## Graphical abstract

Observation of sperm morphology in the testis with scanning and transmission electron microscopy (SEM and TEM).

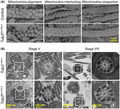

## 1 INTRODUCTION

Spermatozoa are highly specialized cells and consist of a head containing genetic information and a tail that moves vigorously to reach eggs. The sperm tail can be divided into three parts, midpiece, principal piece, and endpiece.^1^, ^2^ An axoneme, a motility apparatus that is composed of “9+2” microtubule arrangement, is found throughout the tail and each tail segment is classified according to the accessory structures surrounding the axoneme. The midpiece contains spirally arranged mitochondria called the mitochondrial sheath and outer dense fibers (ODFs) while the principal piece possesses the fibrous sheath and ODFs. The endpiece contains no accessory structures. Abnormal formation of these structures during spermatogenesis could lead to impaired sperm motility and male infertility.

Tubby family proteins are characterized by the tubby domain in the C‐terminus and are evolutionarily conserved in animals and plants.^3^, ^4^ In mammals, there are five tubby family proteins, TUB, TULP1, TULP2, TULP3, and TULP4. TUB was identified first among tubby family proteins, whose mutation results in obesity, retinal degeneration, and hearing loss in mice.^5^, ^6^ *Tulp1* and *Tulp3* knockout (KO) mice have been generated as well with *Tulp1* KO mice exhibiting retinal degeneration^7^ and *Tulp3* KO mice being embryonic lethal.^8^ Further analyses indicate that TULP3 functions as an adapter protein for ciliary trafficking of membrane‐associated proteins such as G protein‐coupled receptors^9^, ^10^ and nephron‐specific *Tulp3* conditional KO mice exhibited cystic kidneys.^11^ TULP1 is also suggested to be involved in intraflagellar transport in photoreceptor cells.^12^

Unlike other TULP family proteins, *Tulp2* is expressed predominantly in the mouse and human testes.^13^, ^14^ *Tulp2* KO mice were generated recently and exhibited male infertility due to abnormal sperm tail formation.^14^ In this study, we generated *Tulp2* KO mice using not only the conventional method that utilizes homologous recombination in embryonic stem (ES) cells but also the clustered regularly interspaced short palindromic repeats/CRISPR‐associated protein 9 (CRISPR/Cas9) system and observed their defective tail formation in the testes using scanning and transmission electron microscopy (SEM and TEM).

## 2 MATERIALS AND METHOD

### 2.1 Animals

Mice were purchased from CLEA Japan, Inc. (Tokyo, Japan) or Japan SLC, Inc. *Tulp2* KO mice generated in this study are being processed for deposition to the RIKEN BioResource Research Center, Ibaraki, Japan, or the Center for Animal Resources and Development (CARD), Kumamoto University, Kumamoto, Japan. All animal experiments were approved by the Institutional Animal Care and Use Committee of Osaka University (Osaka, Japan) (#Biken‐AP‐H30‐01).

### 2.2 Generation of *Tulp2* KO mice using the conventional method

A 4.0‐kb NotI–XhoI fragment as a short arm and a 5.5‐kb AscI–MfeI fragment as a long arm were obtained by polymerase chain reaction (PCR) using genomic DNA derived from C57BL/6N mice as a template. The PCR primers used are shown in Supplementary Table S1. Both arms were inserted into a modified pNT1.1 vector (www.ncbi.nlm.nih.gov/nuccore/JN935771). The linearized targeting vector was electroporated into EGR‐G101 [(CAG/Acr‐EGFP) B6N × (CAG/Acr‐EGFP) B6N] or EGR‐G01 [129S2 × (cag/acr‐EGFP) B6] ES cells^15^ and colonies were screened. The homologously recombined ES cells were injected into ICR eight‐cell embryos. The embryos were then incubated in a potassium simplex optimized medium (KSOM)^16^ until the next day and transferred into the uterus of pseudopregnant females. The obtained chimeric mice from EGR‐G101 or EGR‐G01 ES cells were mated with C57BL/6N or B6D2F1 females, respectively, for germline transmission. One line was obtained from EGR‐G101 (C57BL/6N‐Tulp2<tm1Osb>/1B) and two lines were obtained from EGR‐G01 (STOCK‐Tulp2<tm1Osb>/3B and STOCK‐Tulp2<tm1Osb>/4A). STOCK‐Tulp2<tm1Osb>/4A line was used for the analyses. Primers used for genotyping are shown in Supplementary Table S1.

### 2.3 Generation of *Tulp2* KO mice using the CRISPR/Cas9 system

Knockout mice were generated using the CRISPR/Cas9 system, as described previously.^17^ The guide RNAs (gRNAs) with fewer off‐target sites were designed with the online software CRISPRdirect.^18^ The gRNA target sequences are shown in Supplementary Table S1. KO mice were maintained on a B6D2 background. Primers used for genotyping are shown in Supplementary Table S1.

### 2.4 Fertility analysis of *Tulp2* KO lines

Sexually mature wild‐type (WT) or *Tulp2* KO male mice were caged individually with three seven‐week‐old female mice for at least five weeks. Male mice were removed after the mating period and females were kept for another three weeks to count the final litters. The numbers of pups and copulation plugs were counted every morning.

### 2.5 Analysis of morphology and motility of spermatozoa

Spermatozoa from cauda epididymis were suspended in Toyoda, Yokoyama, Hoshi (TYH) medium^19^ and placed on MAS‐coated glass slides (Matsunami Glass) for morphology assessment using an Olympus BX53 microscope. For analyzing sperm motility, spermatozoa were incubated in TYH medium for 10 or 120 min and placed in glass chambers (Leja). Sperm motility was analyzed as previously described^20^ using the CEROS II sperm analysis system (software version 1.5; Hamilton Thorne Biosciences).

### 2.6 Histological analysis of testes

Testes were fixed in Bouin's fluid (Polysciences, Inc.) overnight at 4°C, dehydrated by increasing ethanol concentrations, followed by xylene, and embedded in paraffin. Paraffin blocks were sectioned at 5‐μm thickness. The sections were rehydrated and treated with 1% periodic acid for 5 min at room temperature. The sections were then incubated with Schiff's reagent (FUJIFILM Wako Pure Chemical) for 10 min at room temperature, stained with Mayer's hematoxylin solution (FUJIFILM Wako Pure Chemical) for 1 min, and observed with a BX53 microscope (Olympus).

### 2.7 Ultrastructural analysis using transmission electron microscope (TEM)

Testis and cauda epididymis samples were prepared, as previously described.^21^ The ultra‐thin sections were observed using a JEM‐1400plus electron microscope (JEOL) at 80 kV with a CCD Veleta 2K × 2K camera (Olympus).

### 2.8 Ultrastructural analysis using scanning electron microscope (SEM)

Testis samples were prepared with the freeze‐fracture method, as described previously.^21^, ^22^ The samples were observed with an S‐4800 field emission scanning electron microscope (FE‐SEM) (Hitachi).

### 2.9 Statistical analyses

Statistical differences were determined using Welch's *t*‐test by Microsoft Office Excel (Microsoft Corporation). Differences were considered statistically significant if the *p* values were <0.05 (*), 0.01 (**), or 0.001 (***). Data represent the mean ± standard deviation (SD).

## 3 RESULTS

### 3.1 Generation of *Tulp2^tm1^* ^/^ *^tm1^* mice and the results of fertility test

**FIGURE 1:**
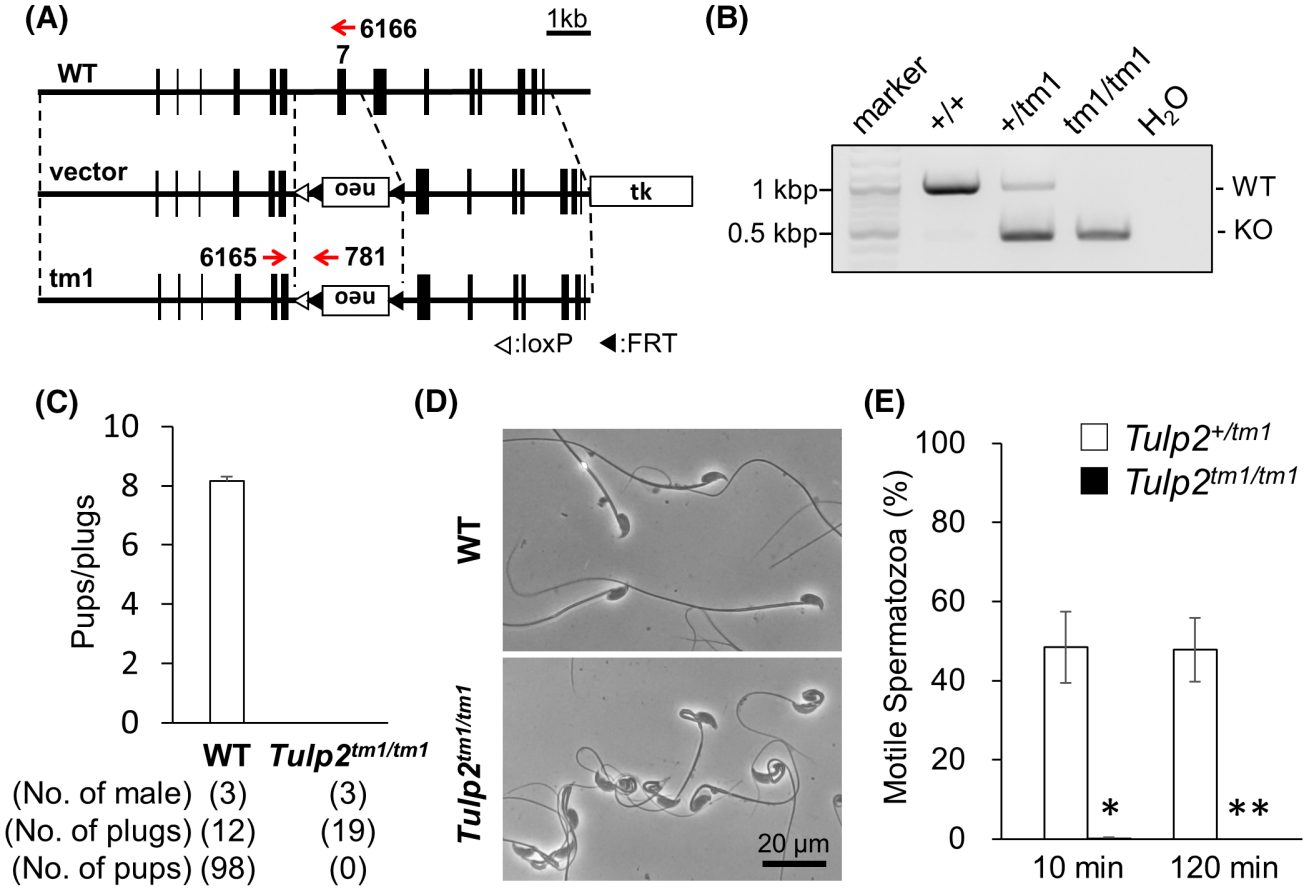
Generation and analyses of *Tulp2^tm1^* ^/^ *^tm1^* mice. (A) Targeting scheme for generating *Tulp2^tm1^* ^/^ *^tm1^* mice. Exon 7 was replaced with a neomycin resistance cassette (neo). Thymidine kinase (tk) was used for negative selection. (B) Genotyping of *Tulp2^tm1^* ^/^ *^tm1^* mice. Primers shown in A were used (#6165 and #6166 for wild type (WT); #6165 and #781 for knockout (KO). Water (H_2_O) was used as a negative control. (C) Number of pups born per plug detected. (D) Observation of spermatozoa obtained from cauda epididymis. (E) Sperm motility was analyzed 10 and 120 min after incubation in a capacitation medium. Number of males analyzed = 3 each

To investigate the function of *Tulp2* in vivo, we generated KO mice (*Tulp1^tm1^* ^/^ *^tm1^* mice) using homologous recombination in ES cells and subsequent generation of chimeric mice. Exon 7 (188 bp) of *Tulp2*, the longest coding exon among exons whose nucleotide length is not divisible by three, was replaced by a neomycin resistance gene cassette (neo) (Figure 1A). *Tulp2^tm1^* ^/^ *^tm1^* mice were obtained by germline transmission and subsequent mating, which was confirmed by genomic PCR (Figure 1B) using primers shown in Figure 1A. *Tulp2^tm1^* ^/^ *^tm1^* mice were viable and showed no overt abnormalities.

To analyze the fertility of *Tulp2^tm1^* ^/^ *^tm1^* males, the mice were caged with three WT females for more than five weeks. Although vaginal plugs were observed 19 times, no pups were obtained from *Tulp2^tm1^* ^/^ *^tm1^* males (Figure 1C), indicating that *Tulp2* is essential for male fertility.

### 3.2 Analyses of sperm morphology and motility in *Tulp2^tm1^* ^/^ *^tm1^* mice

To analyze the cause of male infertility in *Tulp2^tm1^* ^/^ *^tm1^* males, we observed testis sections with a light microscope, but no apparent differences were found between WT and *Tulp2^tm1^* ^/^ *^tm1^* mice (Supplementary Figure S1). We then observed spermatozoa obtained from the cauda epididymis. In *Tulp2^tm1^* ^/^ *^tm1^* males, spermatozoa exhibited abnormal bending or coiling in the midpiece (Figure 1D). Furthermore, motile spermatozoa were rarely observed in *Tulp2^tm1^* ^/^ *^tm1^* males (Figure 1E) using a computer‐assisted sperm analysis (CASA) system. These results suggest that male infertility in *Tulp2^tm1^* ^/^ *^tm1^* mice is caused by abnormal sperm morphology and motility.

### 3.3 Generation and analyses of *Tulp2^em1^* ^/^ *^em1^* mice

**FIGURE 2:**
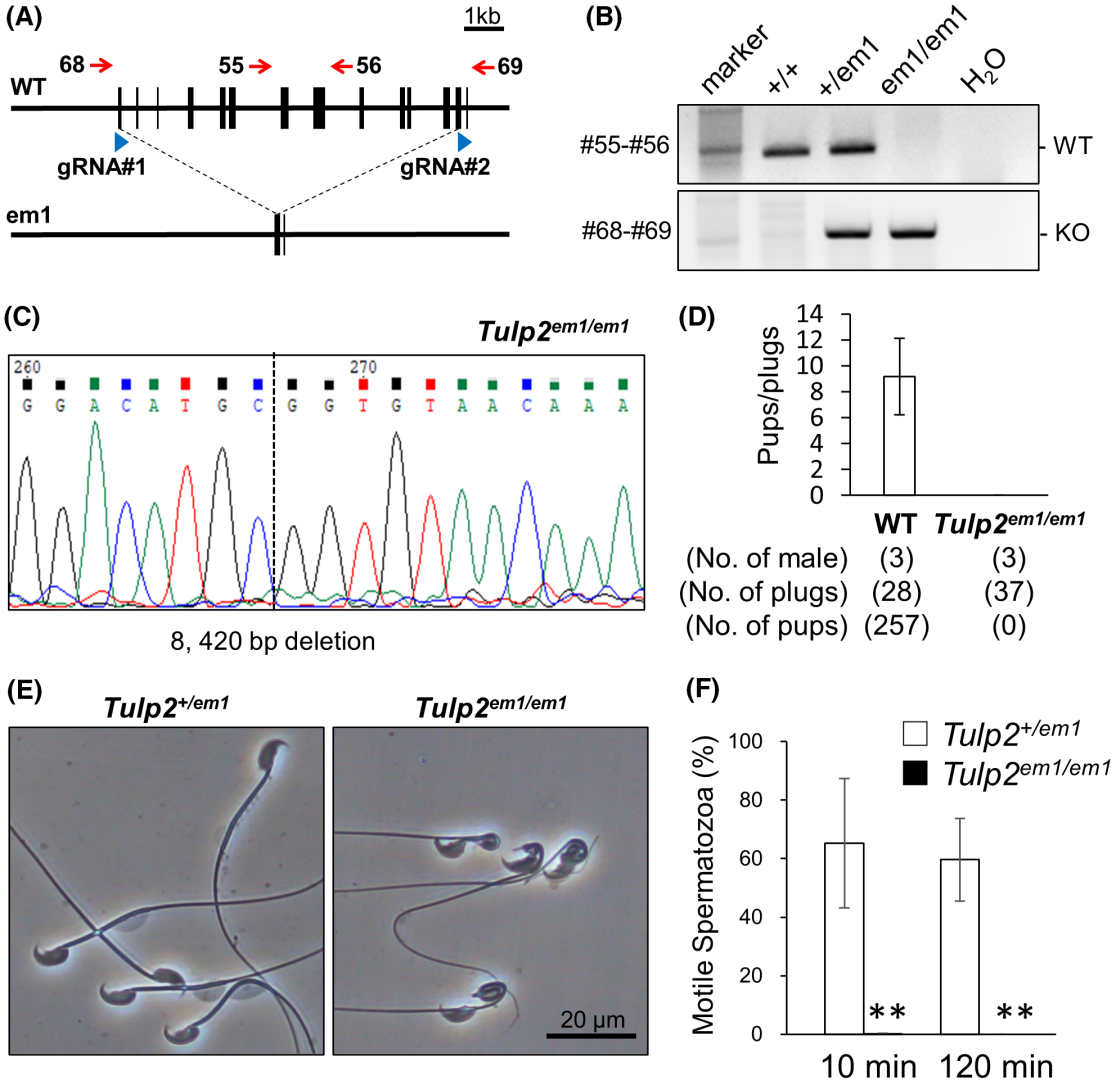
Generation and analyses of *Tulp2^em1^* ^/^ *^em1^* mice. (A) Clustered regularly interspaced short palindromic repeats/CRISPR‐associated protein 9 (CRISPR/Cas9) targeting scheme. Two guide RNAs (gRNAs) were designed to delete almost the entire open reading frame of *Tulp2*. (B) Genotyping of *Tulp2^em1^* ^/^ *^em1^* mice. Primers shown in A were used (#55 and #56 for wild type (WT); #68 and #69 for knockout (KO)). Water (H_2_O) was used as a negative control. (C) Wave pattern sequence of *Tulp2^em1^* ^/^ *^em1^* mice. (D) Number of pups born per plug detected. (E) Observation of spermatozoa obtained from cauda epididymis. (F) Sperm motility was analyzed 10 and 120 min after incubation in a capacitation medium. Number of males analyzed = 4 each

It is possible that *Tulp2^tm1^* ^/^ *^tm1^* mice still generate functional TULP2 because of alteration in splicing patterns. To confirm that whole deletion of *Tulp2* results in the same phenotypes as *Tulp2^tm1^* ^/^ *^tm1^* mice, we also generated KO mice (*Tulp2^em1^* ^/^ *^em1^* mice) using the CRISPR/Cas9 system that has advanced remarkably in recent years.^23^ We designed two gRNAs to delete almost the entire open reading frame of *Tulp2* (Figure 2A). Ribonucleoprotein complexes that contain the gRNA and CAS9 were electroporated into 85 pronuclear embryos and 60 two‐cell embryos were transferred into two pseudopregnant females the next day. Four out of nine pups had large deletions, and one male was caged with two WT females to obtain the F1 generation. Subsequent matings resulted in a KO mouse with an 8,420 bp deletion, which was confirmed by PCR (Figure 2B) and Sanger sequencing (Figure 2C). No overt abnormalities were found in the *Tulp2^em1^* ^/^ *^em1^* mice.

*Tulp2^em1^* ^/^ *^em1^* males were caged with three females for more than eight weeks. Although vaginal plugs were observed 37 times, no pups were obtained from *Tulp2^em1^* ^/^ *^em1^* males (Figure 2D). Furthermore, as consistent with *Tulp2^tm1^* ^/^ *^tm1^* males, spermatozoa of *Tulp2^em1^* ^/^ *^em1^* mice exhibited abnormal morphology in the midpiece (Figure 2E) and almost no motility with CASA (Figure 2F). These results confirm that *Tulp2* is essential for sperm tail formation, motility, and male fertility.

### 3.4 Analyses of sperm morphology in the epididymis

**FIGURE 3:**
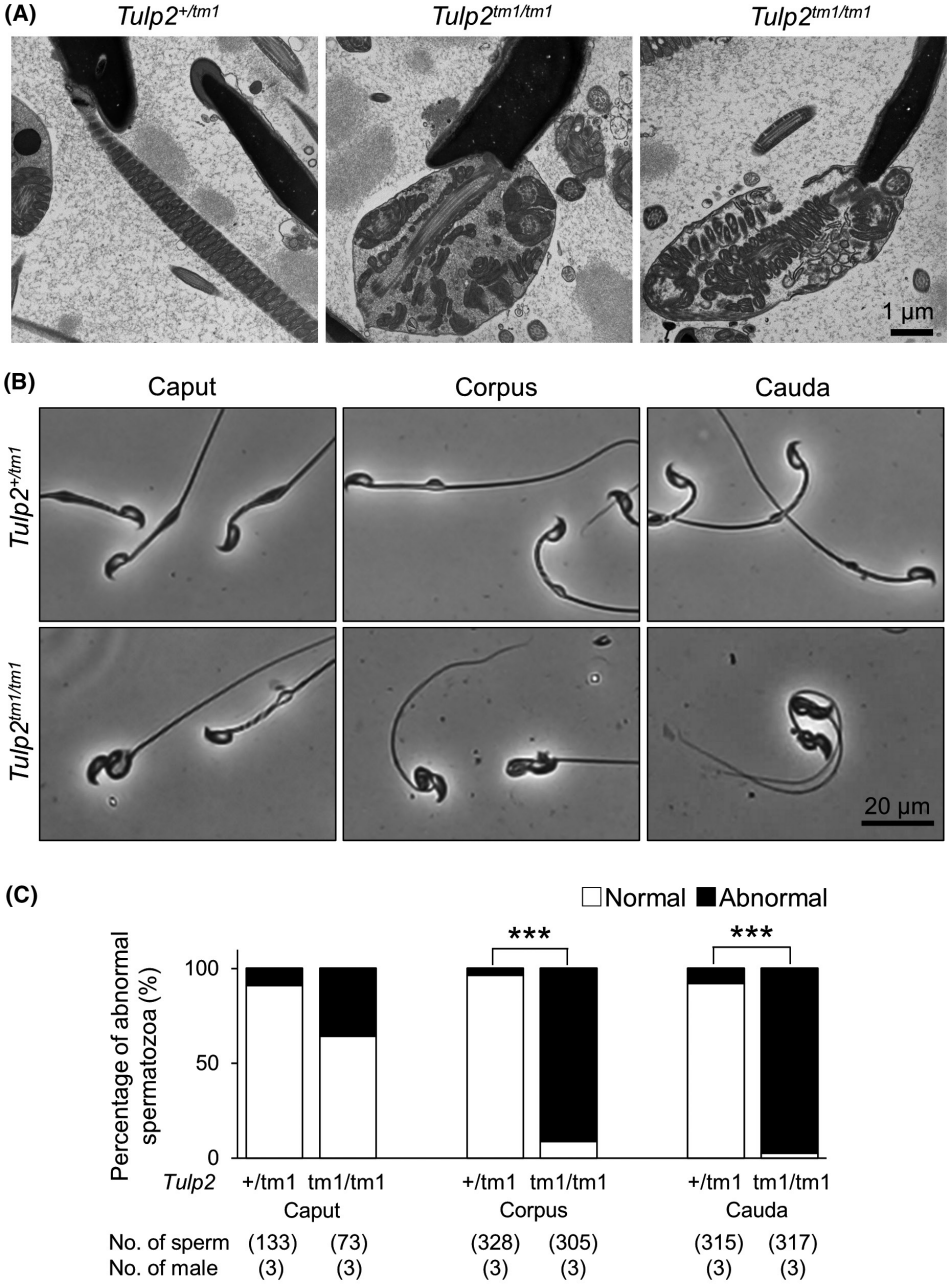
Observation of sperm morphology in the epididymis. (A) Observation of spermatozoa in the cauda epididymis with transmission electron microscopy (TEM). The midpiece was disrupted in *Tulp2^tm1^* ^/^ *^tm1^* mice. (B) Representative images of spermatozoa collected from three sections (caput, corpus, and cauda) of epididymis. (C) Percentages of abnormal spermatozoa collected from the three sections of epididymis

To further analyze sperm morphological abnormalities in *Tulp2^tm1^* ^/^ *^tm1^* mice, we observed spermatozoa in the cauda epididymis using TEM. In the control, mitochondria were helically arranged in the midpiece (Figure 3A). In contrast, the midpiece was disorganized with abnormal mitochondria localization and multiple axonemes enclosed in one plasma membrane in *Tulp2^tm1^* ^/^ *^tm1^* mice (Figure 3A), which is consistent with coiling midpieces observed with light microscopy (Figures 1D and 2E).

To analyze when abnormal midpiece bending or coiling emerged, we observed spermatozoa obtained from caput, corpus, or cauda epididymis. In *Tulp2^tm1^* ^/^ *^tm1^* mice, although almost all spermatozoa exhibited abnormal bending or coiling midpieces in both corpus and cauda epididymis (mean ± SD; corpus = 91.2 ± 5.6%, cauda = 97.5 ± 1.8%), fewer abnormal midpieces were observed in caput epididymis (mean ± SD; 35.6 ± 17.9%) (Figure 3B,C). These results indicate that more bending or coiling of the midpieces occurs during sperm transit in the epididymis, although some spermatozoa showed abnormal midpiece bending or coiling already in the caput epididymis.

### 3.5 Analyses of sperm morphology in the testis

**FIGURE 4:**
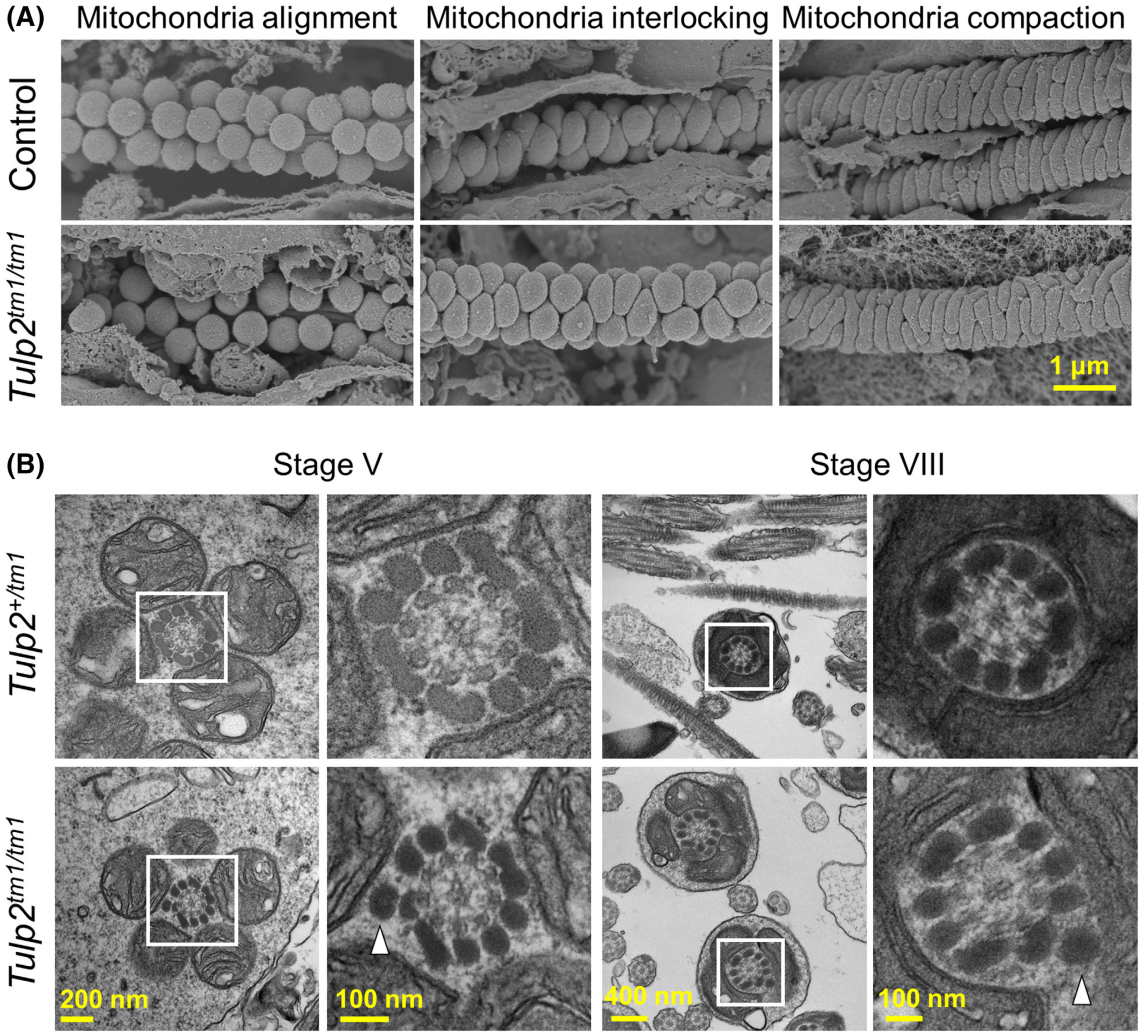
Observation of sperm morphology in the testis. (A) Formation of the mitochondrial sheath during spermatogenesis observed with scanning electron microscopy (SEM). Spherical mitochondria align around the axoneme (left), change their shape in the mitochondrial interlocking step (middle), and form the mitochondrial sheath in the mitochondrial compaction step (right). Wild‐type (WT) and *Tulp2* ^+/^ *^tm1^* mice were used as controls. (B) Observation of spermatids with transmission electron microscopy (TEM). Abnormal extra outer dense fibers (ODFs) are highlighted with white arrowheads. Higher magnification images of the boxed areas are shown to the right

Because abnormal midpieces were already observed in the caput epididymis (Figure 3B,C), we analyzed mitochondrial sheath formation in testes using the freeze‐fracture method with SEM.^21^, ^22^ Unexpectedly, mitochondrial sheath formation (alignment, interlocking, and compaction steps) in *Tulp2^tm1^* ^/^ *^tm1^* mice was comparable to that of control mice (Figure 4A). We then observed spermiogenesis in testis using TEM (stage V and stage VIII). Consistent with the freeze‐fracture method, mitochondrial sheath formation is comparable between *Tulp2* ^+/^ *^tm1^* and *Tulp2^tm1^* ^/^ *^tm1^* mice (Figure 4B). In contrast, we frequently observed an abnormal number of ODFs in *Tulp2^tm1^* ^/^ *^tm1^* mice (number of cross‐sections with extra ODFs; *Tulp2* ^+/^ *^tm1^* = 0 out of 19 sections, *Tulp2^tm1^* ^/^ *^tm1^* = 14 out of 37 sections, all the cross‐sections with extra ODFs showed 10 ODFs, the number of males analyzed = 2 each) (Figure 4B).

## 4 DISCUSSION

By generating two mouse lines of *Tulp2*, we confirmed that *Tulp2* is essential for male fertility. Further analyses indicate that *Tulp2* KO mice exhibited abnormal tail formation and impaired sperm motility. These results are consistent with those of the different mouse line of *Tulp2* KO that was reported recently.^14^ The tail structures of *Tulp2^tm1^* ^/^ *^tm1^* mice were more disorganized during sperm transit in the epididymis (Figure 3B,C). Because *Tulp2* is not expressed in the epididymis,^14^ it is unlikely that TULP2 deletion in the epididymis causes this abnormality. Rather, impaired tail formation in the testis may lead to this phenotype because some spermatozoa exhibited disrupted midpieces already in the caput epididymis (Figure 3B,C). Further morphological abnormalities of midpieces during the epididymal transit have also been reported in the KO mice of other genes such as *Gk2* and *Armc12*.^21^, ^22^

Because the mitochondrial sheath was disrupted in the caput spermatozoa of *Tulp2^tm1^* ^/^ *^tm1^* mice (Figure 3B,C), we observed the formation of mitochondrial sheath during spermatogenesis using the freeze‐fracture method with SEM; however, no overt abnormalities were found in *Tulp2^tm1^* ^/^ *^tm1^* mice (Figure 4A). In contrast, we observed numbers of extra ODFs with TEM (Figure 4B). Because of this abnormal extra ODF, sperm tail structures including mitochondria sheath may be unstable and become disrupted during the later stages of spermatogenesis and sperm transit in the epididymis. Supporting this idea, extra ODFs and abnormal mitochondrial sheath structures were also observed in *Cfap44* KO mice^24^ and infertile patients with *CFAP58* loss‐of‐function variants.^25^ Because it is suggested that CFAP44 (cilia‐ and flagella‐associated protein 44) plays a role in the connection between ODFs and the axoneme^24^ and CFAP58 (cilia‐ and flagella‐associated protein 58) influences ODF protein transportation,^25^ TULP2 may be involved in these processes.

TULP1 and TULP3 are thought to be involved in intraflagellar transport in cilia.^9^, ^10^, ^11^, ^12^ Because sperm flagella possess similar axonemal structures to cilia, it is tempting to hypothesize that TULP2 plays roles in intraflagellar transport of molecules that are involved in the correct ODF formation. However, a recent study indicates that TULP2 could not rescue impaired intraflagellar transport completely in *Tulp3* KO RPE‐1 cells although TUB could rescue the phenotype,^26^ suggesting that TULP2 may not be involved in intraflagellar transport. However, it is still possible that TULP2 functions in intraflagellar transport of sperm flagella in a mechanism different from TULP3 in RPE‐1 cells. It is also possible that TULP2 functions as a transcription factor as suggested by the function of TUB^27^ or functions as an RNA‐binding protein as recently suggested with the analyses of *Tulp2* KO mice,^14^ which impairment may lead to abnormal transcription and/or translation of molecules associated with ODFs. Although *Cfap58* or *Cfap44* was not listed, a recent study detected 1446 differentially expressed genes between WT and *Tulp2* KO testes using RNA sequencing, some of which were related to the cytoskeleton.^14^

In summary, we reveal that mitochondrial sheath formation is not disrupted, but ODF structure is abnormal in *Tulp2* KO mice, which may lead to impaired sperm motility and male infertility. Further analysis of TULP2 may provide a better understanding of the mechanism that regulates the sperm tail formation and may lead to better treatment for male infertility.

## CONFLICT OF INTEREST

Yuki Oyama, Haruhiko Miyata, Keisuke Shimada, Tamara Larasati, Yoshitaka Fujihara, and Masahito Ikawa declare that they have no conflict of interest.

## HUMAN RIGHTS STATEMENTS AND INFORMED CONSENT

This article does not contain any studies with human subjects performed by any of the authors.

## ANIMAL RIGHTS

All institutional and national guidelines for the care and use of laboratory animals were followed.

## APPROVAL BY ETHICS COMMITTEE

All animal experiments performed in this study were approved by the Institutional Animal Care and Use Committee of Osaka University (Osaka, Japan) (#Biken‐AP‐H30‐01).

## Supporting information

Supplementary Information
