# Supplementary material for: TULP2 deletion mice exhibit abnormal outer dense fiber structure and male infertility
Source: Reprod Med Biol. 2022 May 23;21(1):e12467. doi: 10.1002/rmb2.12467 (PMC9126596; doi:10.1002/rmb2.12467)
Supplement: Supplementary file 1 — Supplementary Information [file RMB2-21-e12467-s001.docx]

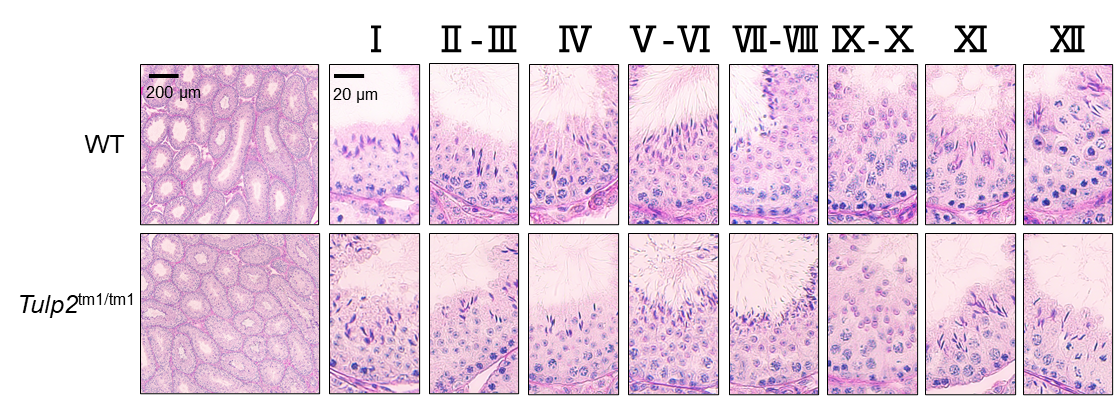


**Supplementary Figure 1. Histological analyses of** ***Tulp2^tm1/tm1^* testis.**

PAS staining of seminiferous tubules of adult mice. No apparent differences were found between WT and *Tulp2^tm1/tm1^* testis*.*

**Supplementary Table 1. Primers and gRNAs used in this study.**

| Method | Target | Strand | Sequence (5'→3') | Name in Fig |
| --- | --- | --- | --- | --- |
| Targeting vector | *Tulp2* tm1 short arm | F | TGACTCCATGGAAAAGATATCTGTCTTAGTC |  |
|  |  | R | ACTATAACAGACTAGACACCCTTACGGATGG |  |
|  | *Tulp2* tm1 long arm | F | TCTACGAGGTTGTAAACTTGACCAGTCAAC |  |
|  |  | R | AAGCTTTCTCCAAACGCACAGAACAG |  |
| Genotyping | *Tulp2* tm1 | F | GGACTCATATTCCATCCGTAAGGGTGTC | 6165 |
|  |  | R | CTCTGGCATCCGTGGATCCAACC | 6166 |
|  |  | R | GCTTGCCGAATATCATGGTGGAAAATGGCC | 781 |
|  | *Tulp2* em1 (KO allele) | F | TACAGGCACTGGCCCCTGGG | 68 |
|  |  | R | TGCGCGTGCTTTGTCATCCC | 69 |
|  | *Tulp2* em1 (WT allele) | F | CTCCCAGGTGTTGGGAGCCC | 55 |
|  |  | R | CCAAATTACCGGGTCCTGGG | 56 |
| gRNA | *Tulp2* em1 |  | TGTTGGACATGGACCGTGAG | gRNA#1 |
|  |  |  | TGGTAAGGGTACCGCGAGTG | gRNA#2 |
